# A mathematical model of honey bee colony dynamics to predict the effect of pollen on colony failure

**DOI:** 10.1371/journal.pone.0225632

**Published:** 2019-11-22

**Authors:** Shahin Bagheri, Mehdi Mirzaie

**Affiliations:** Department of Applied Mathematics, Faculty of Mathematical Sciences, Tarbiat Modares University, Jalal Ale Ahmad Highway, Tehran, Iran; Arizona State University & Santa Fe Institute, UNITED STATES

## Abstract

The decline in colony populations of the honey bee, known as the Colony Collapse Disorder (CCD), is a global concern. Numerous studies have reported possible causes, including pesticides, parasites, and nutritional stress. Poor nutrition affects the immune system at both the individual and colony level, amplifying effects of other stress factors. Pollen is the only source of ten amino acids that are essential to honey bee development, brood rearing and reproduction. This paper presents a new mathematical model to explore the effect of pollen on honey bee colony dynamics. In this model, we considered pollen and nectar as the required food for the colony. The effect of pollen and nectar collected by foragers was evaluated at different mortality rates of pupa, pollen and nectar foragers.

## Introduction

Pollination plays an important role in the ecosystem and drives the evolutionary divergence of plants [1]. The western honey bee (*Apis mellifera* Linnaeus) is the most important pollinator of fruits and vegetable crops in the world [2]. A honey bee colony gathers nectar and pollen from the local environment to produce honey and provide a food supply for its growing population. Colony Collapse Disorder (CCD), a cryptic mass colony death without any clear causal factor, is a phenomenon whereby the majority of worker bees in a colony disappear, yet plenty of food, a few nurse bees, and the queen remain [3]. In the period between 2007 and 2011, about 30% of bees died in the USA due to CCD. Although a single cause for CCD has not been identified, many scientists believe that it may be caused by several possible sources, such as pesticides [4], viruses [5], fungal diseases [6], mite infections [7], nutritional stress [8], and stress from long-distance transportation [9, 10]. It has been reported that interaction between multiple stressors could lead to synergistic effect on mortality rate of honey bees [11, 12].

Nectar, as a major energy source and pollen as a source of protein, vitamin, and lipid, collected by worker bees, are the natural food sources for honey bees. Nectar is converted into honey and stored in honeycombs within the hive to preserve a stable food for winter, while pollen is fed to developing larvae and nurse bees [13]. Although flowers often contain both pollen and nectar, some flowers do not produce nectar (e.g., some wind-pollinated plants). In addition, in some cases, forager bees are specialized in a type of forage, collecting either nectar or pollen from any given plant, even though both are available [14, 15]. Colony and environmental conditions, such as the adult bees and broods population, seasonal changes [16], individual differences [14] and preferences in sensory responsiveness [15, 17], affect forage type collected by bees. Recruiting more pollen foragers increases the collected pollen, allows frequent nursing and leads to higher larval survival and consequently, increases the worker bees in the future [18–20].

Poor nutrition affects the immune system at both individual [8] and colony-level [21], amplifying effects of other stresses. Alaux et al. showed that a shortage of available floral resources directly affects honey bee individual health [8]. Pollen is the only source of ten amino acids that are essential to honey bee development, brood rearing and reproduction; however, these amino acid contents in nectar are negligible [22]. Require et al. reported that a shortage of pollen leads to a reduction in brood production and affects the adult population size and honey reserves [21]. Haydak showed that a lack of pollen in the colony could lead to consuming excess eggs, low brood production, high mortality of worker bees and lack of interest in queen’s care, ultimately causing dangerous problems to the colony [23–25]. Experimental research on honey bee at the colony level is expensive and time-consuming, especially when multiple factors and their interactions affecting the colony are studied [26]. Mathematical modeling allows us to test and analyze the effects of a variety of factors and interactions between them in a fast and cost-effective way [27]. In this study, we develop a mathematical model to predict how honey bee nutrition, by looking specifically at pollen, could affect the honey bee colony dynamics.

Varroa mite, as a primary cause of colony collapse disorder [28], mainly feeds and reproduces on larvae and pupae in the developing brood, leading to genetic defects such as useless wings and weakening the bee by sucking fat bodies of the honey bee [29]. Recent studies have shown that pollen can reduce the effects of Varroa mite [30] and Nosema infection [31]. Therefore, pollen plays a significant role in maintaining the colony’s health and growth [32].

Several mathematical models using differential equations have been proposed to predict and analyze the main factors in the honey bee colony dynamics under specific conditions [33–38]. Khoury et al. [33] introduced a compartment model to analyze the impact of the forager death rate on colony growth. In 2013, they developed their model to include the effect of food availability on colony growth and development [34]. This basic model was extended in later studies by Russel et al. [38], Betti et al. [37], Perry et al. [36] and Paiva et al. [35]. Russel et al. added external factors such as seasonal changes and food availability to determine seasonal colony cycles [38]. Betti et al. combined the dynamics of the spread of disease within a bee colony, taking into account the underlying demographic dynamics of the colony and assessed the ultimate fate of the colony under different scenarios [37]. Perry et al. and Paiva et al. considered the effect of supplemental and artificial feeding on the hive population [35, 36].

Schmickl and Crailsheim [39] constructed one of the most detailed population models (HoPoMo) of honey bee colony dynamics consisting of 60 equations to track every day in the life of a bee from egg to adult bee. The model considered the effect of seasonal changes in egg-laying rate, nurse bees on larvae survival and shortage of pollen on cannibalizing. Adult bees were partitioned into nurse bees, pollen forager, nectar processing bees, and nectar forager. Their model is grounded on the idea of a ‘common stomach’, that relates the division of labor of honey bee colony to colony need [40–46]. Becher et al. developed a dynamical model, BEEHAVE, which combines colony dynamics with foraging patterns and varroa mite dynamics [27]. Booton et al. presented a mathematical model to investigate the effect of external stress on the social inhibition, forager recruitment rate and the laying rate of the queen [47].

In the current research, we developed a compartment model based on Khoury et al. [34] that takes into account the effect of the pollen on colony dynamics. The natural food of honey bee consists of pollen, nectar, and water. Here, we consider only pollen and nectar. The pollen collection can affect survival or colony collapse, and in turn, pollen flux through the colony can influence the size of the brood population. Seasonal changes that affect the amount of food collected by foragers are considered in the proposed model. The model we presented here offers a simple theoretical framework to explore how the dynamics of pollen flow through a colony might interact with population dynamics to determine colony growth. In the following sections, a detailed description of the proposed model is presented, followed by simulation results, and concluding remarks.

## Methods and model

### Basic assumptions

**Fig 1:**
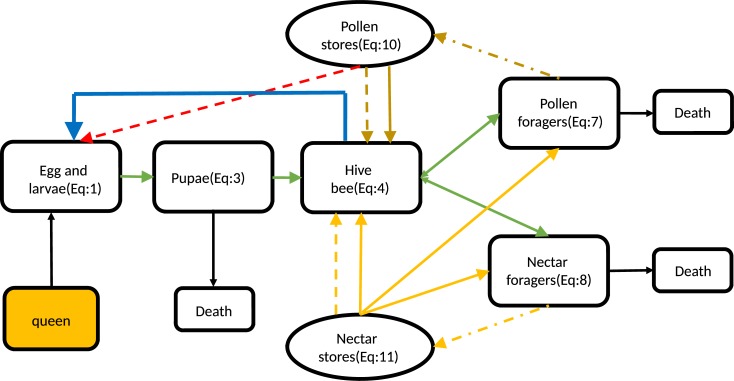
Schematic representation of the presented model. The green lines represent the development stages in the honey bee life cycle. The yellow dash-dotted line represents collected nectar by nectar foragers and the brown dash-dotted line represents collected pollen by pollen foragers. The yellow and brown solid lines represent the consumption of nectar and pollen by adult bees, respectively. The yellow and brown dashed lines represent the consumption of nectar and pollen by nurse bees to feed the larvae. The red dashed line represents the cannibalization. The blue line represents the impact of hive bee numbers on brood survival.

Honey bees have four main development stages in their life cycle: egg, larva, pupa, and finally an adult. In a honeybee colony, a single queen is capable of laying up to 2,000 eggs per day [48]. There are three types of adult bees in a hive, including a queen, workers (female bees) and drones (male bees). Since males (drones) typically comprise less than 5% of the colony in specific seasons and do not contribute to the foraging and colony work [49, 50] they have little impact on colony dynamics and can be ignored [35]. Similar to assumptions by Khoury et al. [34], worker bees are divided into two parts, young and older worker bees. Young worker bees, called hive bees or nurse bees, clean the hive and feed the larvae. They follow a transition period, leave the hive to start foraging duties, and usually forage until their death. If the number of forager bees is higher than required, the behavioral maturation of hive bees will be regulated by a pheromone, ethyl oleate, produced by the foragers. This process is usually referred to as ‘social inhibition’[51]. Similarly, if the number of hive bees is too low, it is possible for foragers to revert back to hive bee duties [52]. Older workers, called ‘foragers’, gather nectar, pollen, water and certain sticky plant resins used in hive construction. In the present study, foragers are divided into pollen foragers that take the pollen into the hive and deposit it into the cells and nectar foragers that collect nectar. Pollen foragers were recognized by their large pollen loads since these bees usually do not collect any additional nectar. Returning bees with extended abdomens and without any pollen on their hind legs were regarded as nectar foragers, although a minority of them may have been water collectors[17]. Foraging behavior is heavily dependent on the needs of the colony [19]. In this study, we assume that the amount of nectar and pollen gathered by foragers depends on their availability in the environment and its requirement. Hive bees produce royal jelly by consuming pollen and the collected nectar is consumed by hive bees, foragers, and uncapped broods. The abstract representation of the assumptions is shown in Fig 1.

### Model equations

In the present study, we extended the model of Khoury et al. [34] and used the same notation for the numbers of hive bees by *H*. In our model, foragers were extended into two categories: *F*_*p*_ (the number of pollen forager bees) and *F*_*n*_ (the number of nectar forager bees). Food collected by foragers, also, was divided into the pollen and nectar, which was collected by the pollen and nectar foragers, shown by *f*_*p*_ and *f*_*n*_ in grams, respectively. The number of eggs and larvae (uncapped brood) is indicated by *B*_*o*_ and the number of pupae (capped cells) that changed into new hive bees is indicated by *B*_*c*_. Time is expressed in days. In reality, only larvae consume pollen, but in our model, we have not separated eggs from larvae and consider an average amount over the whole period before pupation as suggested by Khouri et al. [34]. Time is expressed in days.

The following differential equation was used to model the rate of change among the uncapped brood (eggs and larvae) [34]. The rate of change of uncapped broods (eggs and larvae) is as follows:

$$\frac{dB_{o}}{dt}=LS(.)-\phi_{o}B_{o}$$ (1)

where *L* is the number of eggs laid daily by the queen and *S*(.)is a function that models the survival of uncapped broods (eggs and larvae). We assume that it is dependent on the number of hive bees that feed uncapped broods, and the amount of pollen and nectar gathered by foragers. Khoury et al. [34] introduced a survival function as a function of food and hive bee numbers. Hive bees consume pollen to produce royal jelly, which is the queen and larvae food. Since in our model, natural food is divided into pollen and nectar, we extend the survival function, to include the effect of pollen and nectar on colony growth as separate terms. With the above assumption, the survival function was extended to:

$$S(H,f_{p},f_{n})=(\frac{H}{H+v})(\frac{f_{n}}{f_{n}+b})(\frac{f_{p}{}^{2}}{f_{p}{}^{2}+KH})$$ (2)

The first term considers the role of the number of hive bees on the survival function. Since hive bees are workers in a hive to feed the uncapped brood (egg and larvae) and keep them warm to develop properly, the low hive bee number declines uncapped brood survival. When there are sufficient hive bees for uncapped brood-rearing the first term approaches to 1. The parameter *v* controls the effect of the hive bees on uncapped brood survival as discussed by Khoury et. al [34].

The last two terms indicate that uncapped brood survival declines when nectar and pollen are low. The uncapped brood is fed by hive bees with royal jelly, pollen, and nectar. We assume that the more pollen in a hive will increase the survival rate of hive bees, allow frequent nursing of the broods and lead to fewer brood mortality rate. In fact, hive bees consume pollen to produce royal jelly as uncapped brood’s food. The collected pollen is consumed by the youngest of hive bees, however, for simplicity by making the consumption of pollen proportional to the number of hive bees we assumed that hive bees consume pollen until the transition to foragers. The sigmoid form for these terms explains that survival rates increase rapidly when pollen or nectar reach a viable level as discussed by Khoury et al. [34].

The second term in Eq 1, *ϕ*_*o*_*B*_*o*_ is the rate that uncapped broods change to capped brood (pupae) per day. Uncapped broods (egg and larvae) become capped brood (pupae), and we assume that pupation arises at a constant rate proportional to the number of broods.

Most of the protein needed for the colony is provided from pollen, which is required for egg-laying, to reduce cannibalism and feed larvae. The protein needed for eggs and larvae would be enough if the amount of the collected pollen is proportional to the number of hive bees. Older uncapped brood has the highest pollen demand so that worker bees cannibalize the eggs and young larvae to compensate the shortage of pollen supply to regulate pollen demand. The protein obtained from cannibalism enriches the royal jelly, and increase the chance of older larvae surviving to pupation. Therefore we assume that uncapped brood survival mainly depends on keeping a sufficient supply of pollen and so different terms for pollen and nectar are considered here. The role of hive bees in producing the royal jelly presented in the last term. Parameter K indicates the maximum amount of pollen that can be consumed by a hive bee as a food to be saturated. Schmickl and Karsai introduced the parameter K that is the maximum protein that can saturate a nurse bee [16]. There exist approximately 3500 pollen cells in a hive [53] and each pollen cell contains 230 mg of pollen [54]. In a full-grown colony when there is no shortage of nectar and hive bee, we can assume that the first two terms in Eq (2) are equal to 1. Therefore if we consider K = 8, then in a colony with 20000 nurse bees, the last term is approximately 0.80. In fact, we assumed that at most 80% of the eggs will survive. Because external factors such as disease and weather conditions can endanger the health of eggs.

Additionally, hive bees mix the pollen with some nectar to form a mixture called “bee bread” that used to feed the larvae. The second term in Eq(2) indicates the effect of nectar on *S*(.)and parameter *b* defines the rates of convergence to 1 as *f*_*n*_ grows.

In order to account for the rate of change in the number of capped broods (pupae), we defined the following differential equation and added to the model of Khoury et al. [34]. The equation is composed of three terms: the number of uncapped broods that develop into capped broods, the number of pupae that develop into young bees, and the rate of mortality of capped broods, respectively.

$$\frac{dB_{c}}{dt}=\phi_{o}B_{o}-\varphi_{c}B_{c}-m_{c}B_{c}$$ (3)

where *φ*_*c*_*B*_*c*_ is the rate that young bees emerge from pupation per day, and the last term is the rate that capped broods die.

The following differential equation was used to model the rate of change in the number of hive bees that is composed of two terms: the number of the capped broods that develop into young bees and the number of bees recruited to become pollen and nectars foragers.

$$\frac{dH}{dt}=\varphi_{c}B_{c}-HR_{p}(.)-HR_{n}(.)$$ (4)

where *R*_*p*_(.) and *R*_*n*_(.)are recruitment function, representing the proportional rate of hive bees that become pollen and nectar foragers, respectively. The death rate of hive bees is ignored because they are much safer than the external environment bees [33].

We assume that the transition from young bees into foragers is a function of the number of hive bees, foragers, amount of pollen and nectar in the hive that is increased in the shortage of pollen (nectar) and decreased when there are enough foragers in the hive. The pollen recruitment function is as follows:

$$R_{p}(H,F_{p},F_{n},f_{p})=a_{\min-p}+a_{\max-p}(1-\frac{f_{p}{}^{2}}{\left(f_{p}{}^{2}+KH\right)})-\delta (\frac{F_{p}}{F_{p}+F_{n}+H})$$ (5)

where *a*_min−p_ represents the recruitment rate when there is enough stored pollen in the hive[34]. The second term expresses that the shortage of gathered pollen (i.e., $\left(1-\frac{f_{p}{}^{2}}{\left(f_{p}{}^{2}+KH\right)}\right)$) in the hive is regulated by increasing pollen foragers recruitment. *a*_max−p_ controls the effect of pollen shortage on the transition to pollen foragers. The last term relates the pollen forager to hive bee transition rate that depends on the proportion of pollen foragers in the adult bee population. This phenomenon is known as social inhibition and *δ* controls the strength of this inhibition [34]. Similar recruitment function was considered to describe the transition from hive bee to nectar foragers:

$$R_{n}(H,F_{p},F_{n},f_{n})=a_{\min-n}+a_{\max-n}(1-\frac{f_{n}}{\left(f_{n}+b\right)})-\delta (\frac{F_{n}}{F_{p}+F_{n}+H})$$ (6)

*a*_min−*n*_ and *a*_max−*n*_ have similar definitions to pollen foragers recruitment.

The rate of change of pollen foragers was calculated as follows:

$$\frac{dF_{p}}{dt}=HR_{p}(.)-m_{p}F_{p}$$ (7)

where the first term represents the hive bees to pollen foragers transition rate and the last term is the rate that pollen foragers die.

Similarly, the rate of nectar foragers was also added to the model as follows:

$$\frac{dF_{n}}{dt}=HR_{n}(.)-m_{n}F_{n}$$ (8)

where the first term is the rate that hive bees become nectar foragers and the last term is the rate that nectar foragers die.

The daily rate of change in stored pollen is modeled by the difference in the amount of food brought to the colony by the pollen foragers and the pollen consumed by hive and larvae. Here we assume that pollen is consumed by hive bees and larvae and hive bees eat pollen until they are recruited.

The pollen collected by pollen foragers is variable throughout the year. The flowering of plant species caused one or two different picks of pollen collected. Paiva et al. introduced a function *μ*(.),0≤*μ*(.)≤1 for accounting variations in the availability of natural food, considering environmental factors such as a shortage of food in winter [35]. In this study, we consider *μ*_*p*_(.)governing the variations in the availability of the pollen in a year as follows:

$$\mu_{p}(t)=0.5(\sin((\frac{\pi t}{180})+\frac{\pi }{2})+2.5)$$ (9)

The equation that describes the rate of variation of stored pollen in a colony is given by:

$$\frac{df_{p}}{dt}=\mu_{p}(.)cF_{p}-\gamma_{B_{o}}B_{o}-\gamma_{H}H$$ (10)

where *c* is the maximum pollen brought in daily to the colony by each pollen forager. The consumption of pollen by brood and hive bees is given by $\gamma_{B_{o}}$ and *γ*_*H*_, respectively.

We assume that the nectar is consumed by adult bees and larvae in the colony. The consumption of nectar by brood and adult bees is given by $\lambda_{B_{o}}$ and *λ*_*A*_ respectively. Therefore, similar to pollen consumption, the following differential equation was used to describe the rate of change in nectar:

$$\frac{df_{n}}{dt}=\mu_{n}(.)cF_{n}-\lambda_{B_{o}}B_{o}-\lambda_{A}(H+F_{P}+F_{n})$$ (11)

where *c* is the maximum nectar brought in daily to the colony by each nectar forager and *μ*_*n*_(.) considers the availability of nectar in a year as follows:

$$\mu_{n}(t)=0.5(\sin(\frac{\pi t}{180})+5.5)$$ (12)

The parameter $\lambda_{B_{o}}$ is the average amount of nectar consumed daily by each brood and *λ*_*A*_ is consumption of stored nectar by hive bees, pollen and nectar foragers represented by *H*, *F*_*P*_, and *F*_*n*_. Different parts of the presented compartment model and their relations are shown schematically in Fig 1. Additionally, the list of all differential equations, functions and a brief description explaining the meaning of each term in the model are summarized in Tables 1 and 2.

**Table 1:** List of all differential equations and a brief description explaining their terms.

Differential Equation	Description	#Equation
$\frac{dB_{o}}{dt}=LS(.)-\phi_{o}B_{o}$	The rate of change among the uncapped brood (egg and larvae), where *L* is the number of eggs laid daily by the queen, *S*(.) is the survival function and *ϕ*_*o*_*B*_*o*_ is the rate that uncapped broods change.	1
$\frac{dB_{c}}{dt}=\phi_{o}B_{o}-\varphi_{c}B_{c}-m_{c}B_{c}$	The rate of change in the number of capped broods (pupae), where *φ*_*c*_*B*_*c*_ is the rate that young bees emerge from pupation per day and m rate that capped broods die.	3
$\frac{dH}{dt}=\varphi_{c}B_{c}-HR_{p}(.)-HR_{n}(.)$	The rate of change in the number of hive bees, where *R*_*p*_(.) and *R*_*n*_(.) are recruitment functions, representing the proportional rate of hive bees that become pollen and nectar foragers, respectively.	4
$\frac{dF_{p}}{dt}=HR_{p}(.)-m_{p}F_{p}$	The rate of pollen foragers, where the first term represents the hive bees to pollen forager transition rate and the second term is the rate that pollen foragers die.	7
$\frac{dF_{n}}{dt}=HR_{n}(.)-m_{n}F_{n}$	The rate of nectar foragers, where the first term is the rate that hive bees become nectar foragers and the last term is the rate that nectar foragers die.	9
$\frac{df_{p}}{dt}=\mu_{p}(.)cF_{p}-\gamma_{B_{o}}B_{o}-\gamma_{H}H$	The rate of variation of stored pollen in a colony, where *c* is the maximum pollen brought in daily to the colony by each pollen forager. The consumption of pollen by brood and hive bees is given by $\gamma_{B_{o}}$ and *γ*_*H*_, respectively and *μ*_*p*_(.) governing the variations in the availability of the pollen in a year.	10
$\frac{df_{n}}{dt}=\mu_{n}(.)cF_{n}-\lambda_{B_{o}}B_{o}-\lambda_{A}(H+F_{P}+F_{n})$	The rate of change in nectar, where *c* is the maximum nectar brought in daily to the colony by each nectar forager and the consumption of nectar by brood and adult bees is given by $\lambda_{B_{o}}$ and *λ*_*A*_ respectively and *μ*_*n*_(.) considers the availability of nectar in a year.	11

**Table 2:** List of all functions and a brief description explaining their terms.

Function	Description	#Equation
$S(H,f_{p},f_{n})=(\frac{H}{H+v})(\frac{f_{n}}{f_{n}+b})(\frac{f_{p}{}^{2}}{f_{p}{}^{2}+KH})$	The survival function	2
$R_{p}(H,F_{p},F_{n},f_{p})=a_{\min-p}+a_{\max-p}(1-\frac{f_{p}{}^{2}}{\left(f_{p}{}^{2}+KH\right)})-\delta (\frac{F_{p}}{F_{p}+F_{n}+H})$	The pollen recruitment function	5
$R_{n}(H,F_{p},F_{n},f_{n})=a_{\min-n}+a_{\max-n}(1-\frac{f_{n}}{\left(f_{n}+b\right)})-\delta (\frac{F_{n}}{F_{p}+F_{n}+H})$	The nectar recruitment function	6
$\mu_{p}(t)=0.5(\sin((\frac{\pi t}{180})+\frac{\pi }{2})+2.5)$	The change of availability of pollen in a year.	8
$\mu_{n}(t)=0.5(\sin(\frac{\pi t}{180})+5.5)$	The change of availability of nectar in a year	12

**Fig 2:**
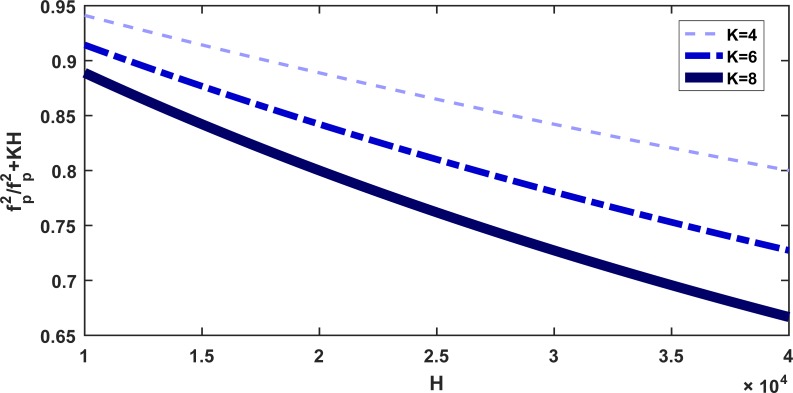
The behavior of $\frac{f_{p}{}^{2}}{f_{p}{}^{2}+KH}$ as a function of H, for fixed values of *f*_*p*_ = 800 and K as mentioned in the legend.

In a hive, there exist approximately 3,500 pollen cells [53], and each pollen cell contains approximately 230mg of pollen [54]. In a colony with 20,000 hive bees that support high-level nursing activity, approximately 800g of pollen is sufficient for the colony to maintain eggs alive [53]. The behavior of $\frac{f_{p}{}^{2}}{f_{p}{}^{2}+KH}$ as a function of H (defined in Eq (2)) is shown in Fig 2. The figure illustrates how the pollen term depends on K at a constant value of *f*_*p*_ = 800. Schmickl et al. [35] showed that when *H* = 10000 and *f*_*p*_ = 800, there is no shortage of pollen in the colony and therefore in the subsequent analysis, we set *K* = 8 as discussed by Schmickl et al. [35].

**Fig 3:**
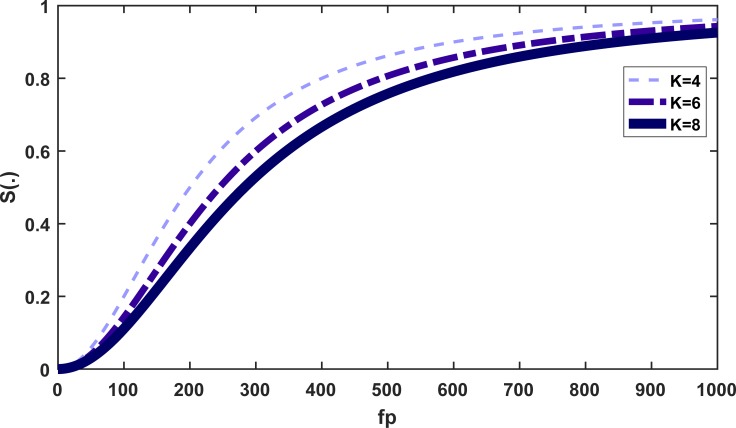
The behavior of *S*(.) as a function of *f*_*p*_ for different values of *K* and constant values of *f*_*n*_, v, b, and H.

Fig 3 shows the behavior of the *S*(.) as a function of *f*_*p*_, for fixed values of *f*_*n*_ = 1000 and *H* = 10000 [34]. By increasing the amount of pollen, the survival function *S*(.) also increases from 0 at *f*_*p*_ = 0 (mg) to 1.

**Fig 4:**
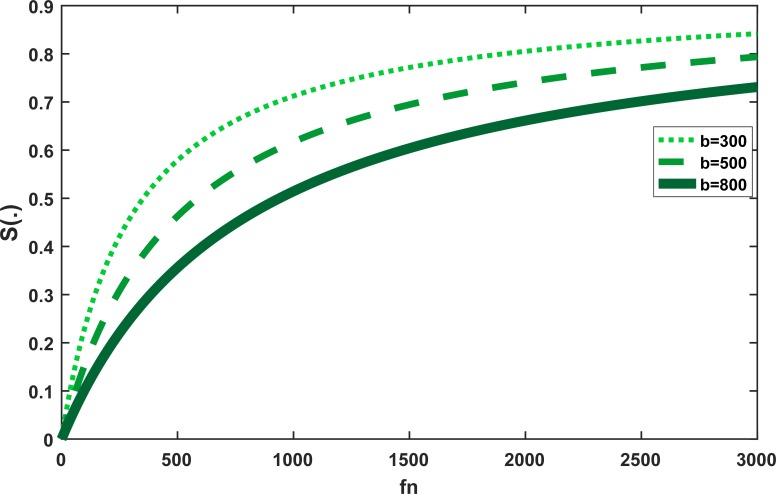
The behavior of *S*(.) as a function of *f*_*n*_ for different values of *K* and constant values of *f*_*p*_, v, b, and H.

Fig 4 shows the behavior of the *S*(*H*,*f*_*p*_,*f*_*n*_) as a function of the amount of stored nectar *f*_*n*_ in the colony. A value of *b* = 500 (g) was chosen as described by Khoury et al. [34].

### Model parameters

The parameters of the model are as important as the equations that were used to construct it. As in [33, 34], we set the daily rate of egg-laying by the queen as L = 2000. Since at least four days (1/*a*_min−*p*_) are required for a hive bee to become a pollen and nectar foragers, *a*_min_ is set to 0.25/day. Additionally, *a*_max−*p*_ is considered equal to *a*_min−*p*_, which indicates doubling the rate of recruitment in the absence of foragers when there is no pollen and nectar in the hive [34]. The similar setting was considered for *a*_max−*n*_ and *a*_min−*n*_. *δ* is set to 0.75 /day, meaning that, when there is no pollen and nectar shortage, pollen and nectar foragers will revert to hive bees if more than one-third of the total bees are foragers. *ϕ*_*o*_ = 1/9*day*^−1^ means that nine days are required for an egg to become a pupa and *ϕ*_*o*_ = 1/12*day*^−1^ means that 12 days are required for a pupa to become a hive bee. Following [33, 34], the maximum amount of food collected daily by each forager is adopted as c = 0.1g. We assume that pollen consumption by each uncapped brood is equal to the average amount of nectar and set to $\lambda_{B_{o}}$ = $\gamma_{B_{o}}$ = 0.018, also *γ*_*H*_ and *λ*_*A*_ was set to 0.007. The list of all model parameters, including their value, references and a brief description of their role is summarized in Table 3.

**Table 3:** List of all model parameters, including their values, references, and a brief description of their role.

Parameter	Description	Value	Ref
*L*	rate of egg-laying by the queen	2000	[33]
*v*	number of hive bees for 50% egg survival	5000	[34]
*b*	mass of nectar stored for 50% egg survival	500	[34]
*a*_min−*p*_	hive bee is recruited to become a pollen forager	0.25	[34]
*a*_max−*p*_	hive bee is recruited to become a pollen forager	0.25	[34]
*a*_min−*n*_	hive bee is recruited to become a nectar forager	0.25	[34]
*a*_max−*n*_	hive bee is recruited to become a nectar forager	0.25	[34]
*δ*	effect of excess foragers on recruitment	0.75	[33]
*ϕ*_*o*_	pupation rate of uncapped brood that changes to pupae per day	1/9	[16]
*φ*_*c*_	pupation rate of capped brood that changes to bee per day	1/12	[16]
$\lambda_{B_{o}}$	daily nectar requirement per uncapped brood	0.018	[34]
$\gamma_{B_{o}}$	daily pollen requirement per uncapped brood	0.018	[34]
*γ*_*H*_	daily pollen requirement per hive bee	0.007	[34]
*λ*_*A*_	daily nectar requirement per adult bee	0.007	[34]
c	food gathered per day per forager	0.1	[34]

## Results and discussion

**Fig 5:**
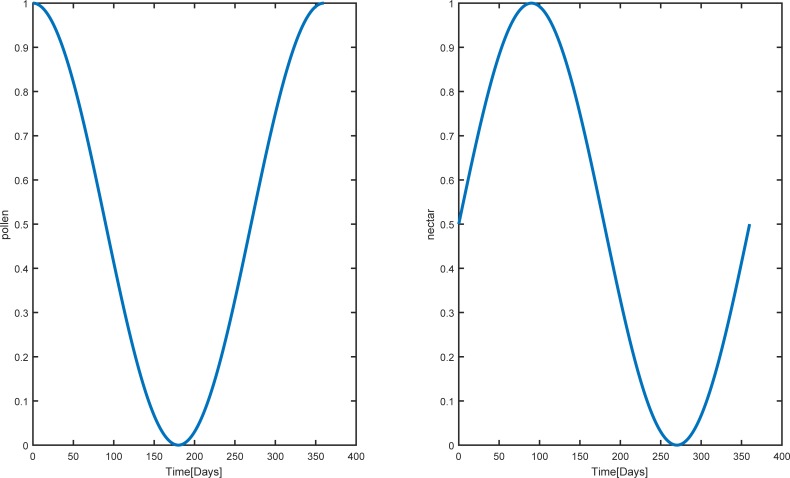
The abundance of the nectar and pollen throughout a year. These hypothetical functions are used for modelling seasonal changes on the dynamics of the colony.

Fig 5 depicts our hypothetical function that relates the abundance of pollen and nectar during a year, beginning from June. We started our simulations with no uncapped and capped brood, 16000 hive bees, 2000 pollen foragers, 6000 nectar foragers, and no pollen and nectar in the colony [55]. The mortality rate of pollen and nectar foragers was set to *m*_*p*_ = *m*_*n*_ = 0.10. The model was implemented for 365 days [34].

**Fig 6:**
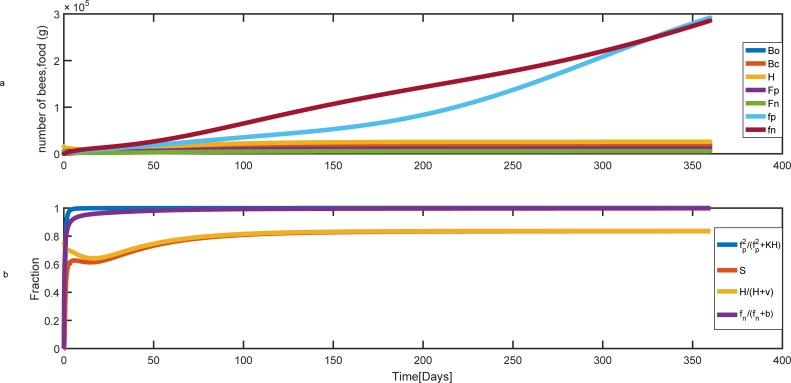
Population and food dynamics over time for the low rate of forager mortality. Parameter values are L = 2000, *γ*_*A*_ *=* 0.007 (gr/day), *γ*_*B*_ *=* 0.018 (gr/day), ν *=* 5000, *a*_min−*p*_ *= a*_min−*n*_ *= a*_max−*p*_ *= a*_max−*n*_ *= 0*.*25 day*^−1^, *δ = 0*.*75 day*^−1^, *φ*_*c*_ = $\frac{1}{12}$ *day*^−1^, *φ*_*o*_ = $\frac{1}{9}$ *day*^−1^, *c = 0*.*1 (gr)*, *m*_*c*_ *= 0*, *K = 8*, *b = 500 (gr)*, *v =* 5000, *m*_*p*_ = *m*_*n*_ *=* 0.1. The hive starts with 16000 hive bees, 2000 pollen foragers, 6000 nectar foragers and no brood, pollen, and nectar at t = 0. (a) Colony population and food behavior during the time. (b) The effect of pollen and the number of hive bees on brood survival. Bo, Bc, H, Fp, and Fn are the number of uncapped broods, capped broods, nurse bees, pollen and nectar foragers, respectively. fp and fn are pollen and nectar stores.

When forager death rates are low, pollen and nectar stores grow rapidly and the reserved food can support the current population and rearing of brood. In this case, the population of honey bee remains constant at a steady-state (Fig 6A) and as shown in Fig 6B, the survival of brood is mainly affected by the number of hive bees.

**Fig 7:**
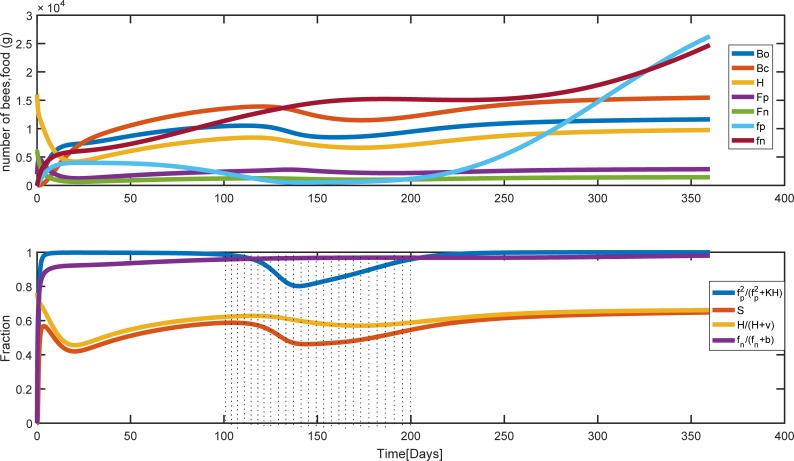
Population and food dynamics for the rate of forager mortality *m*_*p*_ = *m*_*n*_ = *0*.*3*0. Parameter values are the same as Fig 6 except the mortality rates of foragers. (a) Colony population and food behavior during the time. (b) The effect of pollen and the number of hive bees on brood survival. Bo, Bc, H, Fp, and Fn are the number of uncapped broods, capped broods, nurse bees, pollen and nectar foragers, respectively. fp and fn are pollen and nectar stores.

At a higher mortality rate of pollen and nectar foragers, *m*_*n*_ = *m*_*p*_ = 0.30 the equilibrium population size and pollen and nectar stores are decreased when we compared it with *m*_*n*_ = *m*_*p*_ = 0.10 (compare Fig 7A with Fig 6A). The effect of the shortage of pollen on survival function that is modeled using Eq 9 is shown in Fig 7B during t = 100 up to t = 200 (This is from September to January and is dotted-shaded on the figure).

**Fig 8:**
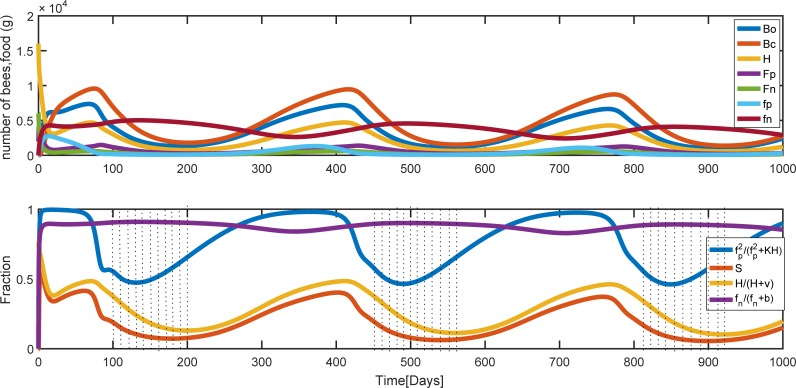
Population and food dynamics for the rate of forager mortality *m*_*p*_ = *m*_*n*_ = 0.42. Parameter values are the same as Fig 6 except the mortality rates of foragers. (a) Colony population and food behavior during the time. (b) The effect of pollen and the number of hive bees on brood survival. Bo, Bc, H, Fp, and Fn are the number of uncapped broods, capped broods, nurse bees, pollen and nectar foragers, respectively. fp and fn are pollen and nectar stores.

At a even higher death rate, *m*_*p*_ = *m*_*n*_ = 0.42 pollen and nectar collected by foragers are decreased, but the colony does not collapse and the nectar remains almost constant with little changes affected by seasonal changes. Since there is not enough pollen forager to gather more pollen than the colony consumes, the amount of stored pollen does not increase but oscillate depending on the seasonal changes. Bee population size is also lower than the previous case. The survival function is also mainly dependent on the pollen, seasonal changes and the number of hive bees (Fig 8). The periods of shortage of pollen are shown by dotted-dashed.

**Fig 9:**
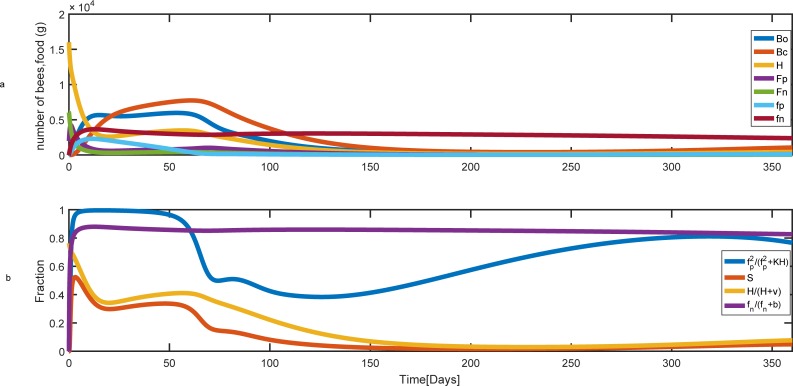
Population and food dynamics for the rate of forager mortality *m*_*p*_ = *m*_*n*_ = 0.50. Parameter values are the same as Fig 6 except the mortality rates of foragers. (a) Colony population and food behavior during the time. (b) The effect of pollen and the number of hive bees on brood survival. Bo, Bc, H, Fp, and Fn are the number of uncapped broods, capped broods, nurse bees, pollen and nectar foragers, respectively. fp and fn are pollen and nectar stores.

By increasing the mortality rate to *m*_*p*_ = *m*_*n*_ = 0.50, the colony will collapse after 150 days, but nectar food remains in the hive even after all bees have died (Fig 9). This may be because the honey bees died before they consume the nectar stores which is completely consistent with the observation of rapid declines in colony collapse disorder along with remaining stored food in the hive [34]. In this case, because of the lack of pollen which is an essential food for brood rearing and reproduction, the adult bees die faster than they are replaced by younger bees. Pollen is needed to ensure that a colony can replace the lost bees.

In summary, our model predicts that different forager death rates lead to different colony behaviors, which range from a persistent population with an excess of pollen and nectar stores, to a stable population with limited pollen and nectar stores, to a collapsed colony with residual nectar stores.

**Fig 10:**
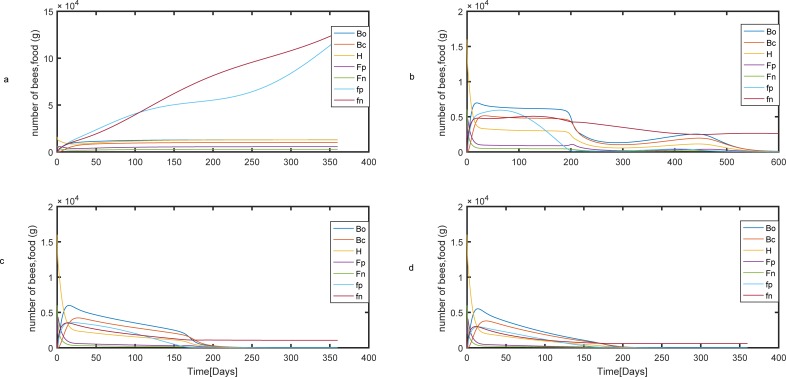
Population and food dynamics for different rates of forager mortality when the mortality rate of the pupa is equal to *m_c_* = 0.06. (a)m_p_ = m_n_ = 0.10. (b) m_p_ = m_n_ = 0.30 (c) m_p_ = m_n_ = 0.42 (d) m_p_ = m_n_ = 0.50. Bo, Bc, H, Fp, and Fn are the number of uncapped broods, capped broods, nurse bees, pollen and nectar foragers, respectively. fp and fn are pollen and nectar stores.

Fig 10 shows results from the model when the mortality rate of the pupa is raised to *m*_*c*_ = 0.06. In Fig 10A, the forager mortality rate is low (m = 0.1), but increasing the pupa death rate leads to a decline in a number of hive bees (compare Fig 10A with Fig 6A). At this case, our model predicts that the colony will survive. At intermediate mortality rate of foragers, the colony collapsed after 600 days and stored food remains in the hive (Fig 10B). Increasing the death rate of foragers leads to a rapid decline in adult bees after 200 days (Fig 10C and 10D).

**Fig 11:**
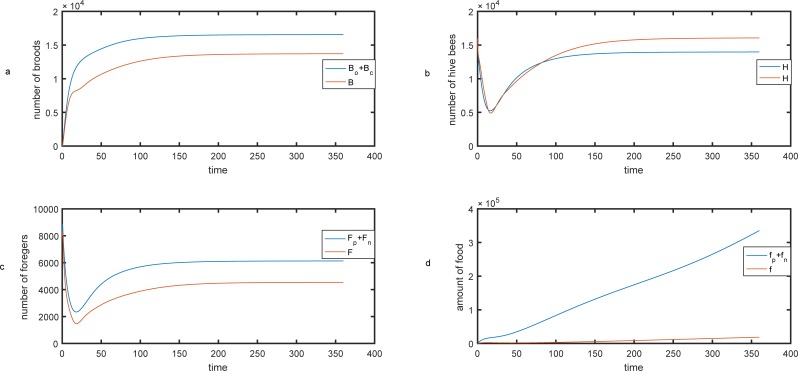
Population dynamics of our model compared to Khoury et al. model for the rate of forager mortality *m*_*p*_ = *m*_*n*_ = 0.30. Dynamics of the a) brood b) hive bee c) foragers population and d) food behavior over time. The blue and red lines are the values of simulated variables in our model and the Khoury et al. model, respectively.

The simulation results of the presented model were compared to Khoury et al. [34] in Fig 11. The blue and red lines are the values of simulated variables in our model and the Khoury et al. model, respectively. Our model is implemented using the following parameters: L = 2000, *λ*_*A*_ = 0.007 (gr/day), $\gamma_{B_{o}}$ = 0.018 (gr/day), *a*_min_*p*_ = *a*_min_*n*_ = *a*_max_*p*_ = *a*_max_*n*_ = 0.25 *day*^−1^, *δ* = 0.75 *day*^−1^, *φ*_*c*_ = $\frac{1}{12}$ *day*^−1^, *ϕ*_*o*_ = $\frac{1}{9}$ *day*^−1^, *c* = 0.10 (gr), *m*_*c*_ = 0, *K* = 8, *b* = 500 (gr), *v = 1000*, *m*_*p*_ = *m*_*n*_ = 0.30.

For Khoury et.al (2011) model the following parameters is used: L = 2000, *γ*_*A*_ = 0.007 (gr/day), *γ*_*B*_ = 0.018 (gr/day), *τ* = 12, *a*_min_ = *a*_max_ = 0.25 *day*^−1^, *σ* = 0.75 *day*^−1^, *ϕ* = $\frac{1}{9}$ *day*^−1^, *c* = 0.10 (gr), *b* = 500 (gr), *v = 5000*, *m* = 0.30.

Fig 11A compares the results of the number of broods in both models. Note that, in our model, the number of broods is equal to the number of uncapped broods (Bo) plus the number of capped broods (Bc). Fig 11B shows the dynamics of the hive bee population throughout the year. The forager bees in our model were divided into pollen and nectar foragers, and also the collected food was dissected to pollen and nectar collected by foragers. Fig 11C and 11D compare the results of both models.

**Fig 12:**
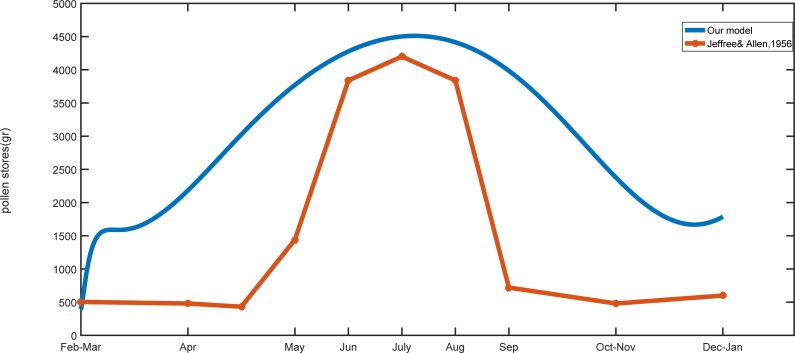
Dynamics of pollen stores in our simulated honey bee colony and empirical data.

Finally, the collected pollen simulated by the model (Eq 10) with parameters *m*_*p*_ = *m*_*n*_ = 0.42 compared with experimental data from Jeffree and Allen [53]. Fig 12 indicates similar behavior of the results of the model and experimental data.

The main purpose of this study is to present a framework that considers the factors that affect the population of the honey bee. The model is based on a dynamical model introduced by Khouri et al. [34]. We have extended the model by dividing food into pollen and nectar and also foragers to pollen and nectar foragers. The modeling framework we present here is a simplification of the real world and was constructed based on assumptions mentioned in the basic assumption section and the results are merely a simulation of the model not reality. One of the main factors that affects the honey bee colony dynamics is weather condition (rain, wind, and drought) that hinders the bees in leaving the hive or plants in producing flowers. The model could be extended to incorporate the effect of weather conditions on nectar or pollen collection. Because there can be periods with poor weather or poor flowering in which honey bees cannot find any nectar and pollen periods.

The honey bee queen needs constant care and supports by young worker bees that consume stored pollen in the hive to produce the protein needed for the queen. Therefore, the egg-laying rate relies on the synergy and consolidated efforts of the queen and the workers in the colony. Fine et al. monitored queen egg-laying under different conditions and showed that pollen nutrition affects it. In this study, for simplicity we assumed that the number of eggs laid daily by the queen is constant and the model could be extended to consider the synergy between queen, workers and the amount of pollen.

Several studies have suggested multiple causes of colony collapse disorder including parasites, pathogens, and pesticides, but Horn et al. [56] discussed the impact of forage availability on colony health. In reality, the period of time in which there is shortage of nectar and pollen affects the honey bee colony. They investigated how honeybee colonies deal with different forage stress factors including overall forage supply, the foraging distance to forage source, and the timing and duration of temporal forage gaps. The model that we have presented here predicts how food availability (nectar and pollen) and forager death rate influence colony growth and development. In our model, the forage availability is abstracted in the mortality rate of foragers and availability of pollen and nectar based on seasonal changes, so the model could be extended to address more details and interactions of forages availability. In the first scenario, under conditions of the low mortality rate of foragers and high food availability, the model predicts that the amount of stored nectar and pollen grows very quickly (Fig 6). This is not consistent with the reality of honey bee behavior, because nectar storage is more important than pollen reserves. In this case more foragers should shift to collect nectar which our model does not consider.
